# Early Intervention vs Conservative Management in Severe Asymptomatic Aortic Stenosis: A Critical Review of Randomized Evidence

**DOI:** 10.1016/j.jacadv.2025.102178

**Published:** 2025-09-19

**Authors:** Victor Dayan, Mateo Marin-Cuartas, Raffaele De Caterina, Suzanne De Waha, Nicolas M. Van Mieghem, Michael A. Borger, Robert O. Bonow, Deepak L. Bhatt

**Affiliations:** a Centro Cardiovascular Universitario, Universidad de la Republica, Montevideo, Uruguay; b University Department of Cardiac Surgery, Leipzig Heart Center, Leipzig, Germany; c Chair of Cardiology, University of Pisa and Cardiology Division 1, Pisa University Hospital, Pisa, Italy; d Department of Interventional Cardiology, Thoraxcenter, Cardiovascular Institute, Erasmus University Medical Center, Rotterdam, Netherlands; e Northwestern University Feinberg School of Medicine, Chicago, Illinois, USA; f Mount Sinai Fuster Heart Hospital, Icahn School of Medicine at Mount Sinai, New York, USA

**Keywords:** asymptomatic severe aortic stenosis, surgical aortic valve replacement, SAVR, transcatheter aortic valve replacement, TAVI, TAVR, transcatheter aortic valve implantation

## Abstract

Aortic stenosis (AS) is a progressive disease that may remain asymptomatic despite underlying myocardial damage. Management of asymptomatic severe AS remains controversial, especially in the current era of safer surgical and transcatheter valve replacement. This critical review examines 4 randomized controlled trials—AVATAR (Aortic Valve Replacement Vs Conservative Treatment in Asymptomatic Severe Aortic Stenosis), RECOVERY (Randomized Comparison of Early Surgery vs Conventional Treatment in Very Severe Aortic Stenosis), EARLY TAVR (Evaluation of TAVR Compared to Surveillance for Patients with Asymptomatic Severe Aortic Stenosis), and EVOLVED (Early Valve Replacement Guided by Biomarkers of Left Ventricular Decompensation in Asymptomatic Patients with Severe Aortic Stenosis)—comparing early aortic valve replacement with conservative management. While early intervention reduces composite endpoints involving heart failure hospitalization, individual trials have not demonstrated consistent mortality or stroke benefits. Importantly, sudden cardiac death was rare across all trials, and close surveillance appeared to be a key determinant of outcomes in the conservative arms. Differences in surveillance intensity, trial populations, and valve types limit pooled interpretations. Current evidence supports a tailored approach: conservative management is reasonable when reliable follow-up can be ensured, while early aortic valve replacement may benefit selected patients. Ongoing trials will help clarify long-term outcomes, optimal timing, and risk stratification strategies in asymptomatic AS.

## Highlights

• Early AVR for asymptomatic AS is increasingly considered as procedural risk declines.
• Trials show fewer hospitalizations but no consistent mortality benefit with early AVR.
• Close clinical surveillance remains essential for safely delaying intervention in selected patients.
• Future studies should define long-term outcomes and refine imaging and biomarker-based surveillance.

Aortic stenosis (AS) is a progressive disease primarily caused by degenerative calcification, bicuspid aortic valve disease, and, less commonly, rheumatic valve disease.^1^ The progressive reduction in effective orifice area leads to increased afterload, resulting in compensatory left ventricular (LV) hypertrophy. The combination of hypertrophy, myocardial fibrosis, and ischemia may explain the development of symptoms as the severity of AS progresses, but the risk of sudden cardiac death (SCD) in asymptomatic patients remains very low in patients who have close follow-up.^2^ However, median survival in those with symptomatic severe AS is only 3 years if left untreated.^3^

Clinical surveillance was traditionally recommended for patients with asymptomatic AS, based on historical data when perioperative risk for surgical aortic valve replacement (SAVR) was higher than observed in contemporary practice and well before the pre-transcatheter aortic valve replacement (TAVR) era. Nonrandomized studies in patients with asymptomatic severe AS and normal left ventricular ejection fraction (LVEF) have reported improved survival associated with early intervention in patients without other indications for AVR, but these studies are prone to selection bias.^4^ Therefore, our objective in this review paper is to provide an analysis of current randomized control trials (RCTs) to critically assist the medical community in the management of asymptomatic patients.

## Evidence from randomized control trials

### Recovery

The RECOVERY (Randomized Comparison of Early Surgery vs Conventional Treatment in Very Severe Aortic Stenosis) trial^5^ evaluated the efficacy of SAVR in patients with very severe AS and LVEF ≥50% (Table 1). Exercise testing was performed selectively in patients with dubious symptoms. The primary outcome was a composite of operative mortality or cardiovascular (CV) mortality during follow-up. A total of 145 patients were randomized and the median follow-up was 74 months. The average age was 64 years and the mean European system for cardiac operative risk evaluation II score was 0.9%. A mechanical prosthesis was implanted in 50% of SAVR patients. Fifty-three patients in the conservative group (74%) underwent SAVR after a median follow-up of 23 months. The operative mortality was 0% in SAVR patients. Patients randomized to early surgery had a significantly lower incidence of the primary composite endpoint (1% vs 15%; HR: 0.09 [95% CI: 0.01-0.67]). The overall mortality was significantly lower in the early surgical group (7% vs 21%; HR: 0.33 [95% CI: 0.12-0.90]). The annual rate of SCD in the conservative group at 4 years was 1%. Both overall and CV mortality showed a marked increase between 6 and 8 years, from 10% to 32% and 6% to 26%, respectively. This suggests that there was less attention to surveillance in later years. With the small number of SCDs, it is possible that patients developed symptoms or progressively severe AS and could have been referred to AVR prior to their deaths.

**Table 1:** Randomized Controlled Trials of Early Intervention Versus Conservative Management in Severe Asymptomatic Aortic Stenosis

Trial	N	Inclusion Criteria	Mean Age (y)	Primary Outcome	LVEF >50% Required	Intervention	AVR at Follow-Up (%) in Cons Arm	Median Follow-Up (y)	Median Time to AVR in Cons (mo)	Annual Mortality Rate (Deaths/[Pop At Risk*Median Follow-Up]*100)	
										AVR	Cons
RECOVERY	145	• AVA ≤0.75 cm^2^ and • Vmax ≥4.5 m/s or mean gradient ≥50 mm Hg	64	Operative mortality or cardiovascular mortality during follow-up	Yes	SAVR (100%)	74	6.2	23	5/(73*6.2) 1.1	15/(72*6.1) 3.4
AVATAR	157	• AVA ≤1 cm^2^ or AVA index ≤0.6 cm^2^/m^2^ and • Vmax >4 m/s or mean gradient ≥40 mm Hg	67	All-cause mortality or MACE (acute MI, stroke, and unplanned HF hospitalization needing intravenous treatment with diuretics or inotropes)	Yes	SAVR (100%)	44	5.3	16	13/(78*5.3) 3.1	27/(79*5.3) 6.4
EARLY TAVR	901	• AVA ≤1.0 cm^2^ or AVA index ≤0.6 cm^2^/m^2^ and • Vmax ≥4.0 m/s or mean gradient ≥40 mm Hg	76	All-cause mortality, stroke, or unplanned hospitalization for cardiovascular causes (including unplanned AVR in first 6 months in Cons arm)	Yes	TAVR (100%)	87	3.8	11	38/(455*3.8) 2.2	41/(446*3.8) 2.4
EVOLVED	224	• Myocardial fibrosis on CMR and • Vmax ≥4.0 m/s or ≥3.5 m/s with an AVA index <0.6 cm^2^/m^2^	76	All-cause mortality or unplanned aortic stenosis-related hospitalization	Yes	SAVR (75%) TAVR (25%)	77	3.5	20	16/(113*3.5) 4.0	14 (111*3.5) 3.6

AVA = aortic valve area; AVR = aortic valve replacement; AVATAR = Aortic Valve Replacement Vs Conservative Treatment in Asymptomatic Severe Aortic Stenosis; CMR = cardiac magnetic resonance; Cons = conservative treatment arm; EARLY TAVR = Evaluation of TAVR Compared to Surveillance for Patients with Asymptomatic Severe Aortic Stenosis; EVOLVED = Early Valve Replacement Guided by Biomarkers of Left Ventricular Decompensation in Asymptomatic Patients with Severe Aortic Stenosis; HF = heart failure; LVEF = left ventricular ejection fraction; MACE = major adverse cardiovascular events; MI = myocardial infarction; RECOVERY = Randomized Comparison of Early Surgery vs Conventional Treatment in Very Severe Aortic Stenosis; SAVR = surgical aortic valve replacement; TAVR = transcatheter aortic valve replacement; Vmax = peak aortic valve jet velocity.

Annual mortality rate was calculated as an actuarial estimate (total deaths/population at risk x median follow-up x 100). Constant hazard was assumed for its calculation considering all included trials reported constant HR for overall mortality report. Data on annual mortality should not be linearly extrapolated to estimate long-term outcomes, as this may lead to underestimation or overestimation of true cumulative mortality.

## Avatar

The AVATAR (Aortic Valve Replacement Vs Conservative Treatment in Asymptomatic Severe Aortic Stenosis) trial^6^ evaluated the safety and efficacy of SAVR in patients with severe AS and LVEF ≥50%. The main inclusion criteria are summarized in Table 1. To confirm the asymptomatic status, all patients underwent an exercise test and were able to achieve 85% of the maximum predicted heart rate without symptoms. The primary outcome was a composite of all-cause death or major adverse CV events, consisting of acute myocardial infarction, stroke, and unplanned heart failure (HF) hospitalization needing intravenous treatment with diuretics or inotropes. Power calculation for the primary outcome called for 312 patients but only 157 patients were randomized, with a median follow-up of 32 months in the original publication.^6^ A single center enrolled 73% of patients. The average age was 67 years, and the mean Society of Thoracic Surgery score was 1.7%. A mechanical prosthesis was implanted in 47.2% of patients. Operative mortality was 1.4% in the SAVR group. Taking into consideration that the trial was underpowered and ended prematurely, patients randomized to early surgery had a significantly lower incidence of the primary composite endpoint (15.2% vs 34.7%; HR: 0.46 [95% CI: 0.23-0.90]; *P* = 0.02). No differences were found in overall mortality.

The authors recently reported their updated results with an extended follow-up of 63 months.^7^ They found a lower incidence of the primary outcome (23.1% vs 46.8%; HR: 0.42 [95% CI: 0.24-0.73]) with early surgery, as well as lower overall mortality in the SAVR group (16.7% vs 34.2%, HR: 0.44 [95% CI: 0.23-0.85]).^7^ Thirty-nine of the 79 patients (49.4%) in the conservative group underwent AVR (35 SAVR and 4 TAVR). The most common indication for AVR in this group was onset of symptoms (51.4%). The annual rate of SCD in asymptomatic patients in the conservative group was 1.48% vs 1% in the early surgery group.

## Early TAVR

The EARLY TAVR (Evaluation of TAVR Compared to Surveillance for Patients with Asymptomatic Severe Aortic Stenosis) trial evaluated the efficacy of TAVR with a balloon expandable valve (SAPIEN 3 or SAPIEN 3 Ultra, Edwards Lifesciences) in patients with severe AS and LVEF ≥50%.^8^ The main inclusion criteria were patients with severe asymptomatic severe AS older than 65 years, and with anatomy suitable for transfemoral TAVR. An exercise test was performed in 90.6% of patients to confirm the asymptomatic status, and the other 9.4% of patients not able to perform a stress test were categorized as asymptomatic based on a detailed physician assessment of medical history. The primary outcome was a composite of death from any cause, stroke, or unplanned hospitalization for CV causes. Any AVR in the clinical surveillance group within 6 months after randomization was deemed an unplanned hospitalization for CV causes and, therefore, a primary outcome event. A total of 901 patients were randomized. The average age was 76 years, and the mean Society of Thoracic Surgery score was 1.8%. In the conservative group, 105 patients (23%) underwent TAVR within 6 months of randomization, and 87% underwent TAVR after a median of 11 months after randomization. The most common indication for intervention in this group was the onset of symptoms. The 30-day mortality after TAVR was 0.9%. Patients randomized to TAVR had a significantly lower incidence of the primary composite endpoint at a median follow-up of 3.8 years. (26.8% vs 45.3%; HR: 0.50 [95% CI: 0.40-0.63]), driven by unplanned AVR within 6 months of randomization in the conservative group. The overall mortality was similar in the 2 groups (8.4% vs 9.2% in the TAVR and conservative arms, respectively, Table 1). SCD was approximately 0.4% and 0.3% per year in the early intervention and conservative groups, respectively. This trial has recently resulted in the U.S. Food and Drug Administration approval of these valves for patients with asymptomatic severe AS (https://www.acc.org/Latest-in-Cardiology/Articles/2025/05/01/16/09/FDA-Update-Agency-Approves-TAVR-For-Asymptomatic-Severe-AS).

## Evolved

The EVOLVED (Early Valve Replacement Guided by Biomarkers of Left Ventricular Decompensation in Asymptomatic Patients with Severe Aortic Stenosis) trial^9^ evaluated the efficacy of AVR (either TAVR or SAVR) in patients with severe AS, LVEF ≥50%, and evidence of myocardial fibrosis. Patients were initially deemed eligible after evidence of LV remodeling either with high-sensitivity troponin I concentration ≥6 ng/L or the presence of LV hypertrophy on the electrocardiogram. Patients thus selected then underwent cardiac magnetic resonance imaging with gadolinium contrast, and randomization was performed only after confirmation of midwall myocardial fibrosis. Exercise testing was not compulsory to define the asymptomatic status. The primary outcome was a composite of all-cause mortality or unplanned AS-related hospitalization. A total of 224 patients were randomized. SAVR and TAVI were performed in 75% and 25% of the early intervention group, respectively. The median follow-up was 42 months, and the average age was 76 years. Eighty-five patients in the conservative group (77%) underwent AVR during the follow-up and 28% did so within 12 months of randomization. Of the patients in the conservative group who underwent AVR, 55% underwent SAVR. The most common indication for AVR in this group was onset of symptoms. The 30-day mortality was 1%. No differences in the primary outcome were found in the early intervention and conservative groups (18% vs 23%, respectively; HR: 0.79 [95% CI: 0.44-1.43]). The overall mortality was similar in the 2 groups (14% vs 13% in early and conservative groups, respectively).

## Interpretation and appraisal of the evidence from current trials

### All-cause death

**Figure 1:**
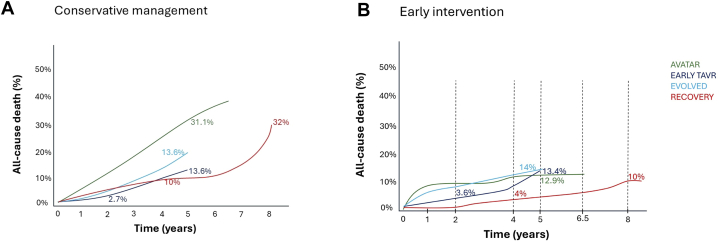
**Schematic Representation of All-Cause Death** (A) Conservative and (B) aortic valve replacement arms of the analyzed trials. Data on mortality across time were extracted from the included studies. EVOLVED = Early Valve Replacement Guided by Biomarkers of Left Ventricular Decompensation in Asymptomatic Patients with Severe Aortic Stenosis; AVATAR = Aortic Valve Replacement Vs Conservative Treatment in Asymptomatic Severe Aortic Stenosis; EARLY TAVR = Evaluation of TAVR Compared to Surveillance for Patients with Asymptomatic Severe Aortic Stenosis; RECOVERY = Randomized Comparison of Early Surgery vs Conventional Treatment in Very Severe Aortic Stenosis.

To understand the potential benefit of early AVR on mortality and its comparison among trials, it is critical to understand the death rate of the conservative group in each trial (Figure 1). Furthermore, mortality analyses in the conservative arms provide an understanding of the real natural-history outcome of asymptomatic AS under actual guidelines to help define the best follow-up strategy in these patients.

Conservative arms: Mortality in the conservative arm is largely related to both the pattern of clinical surveillance and the percentage of patients who cross over to AVR during randomized follow-up. EARLY TAVR was the trial with the highest percentage of AVRs (87%) and the lowest death rate in the conservative arm, while AVATAR was the trial with the lowest percentage of AVR (44%) and the highest mortality in the conservative group (Figure 1). The meta-analysis by Gahl et al^4^ on the natural history of severe AS in asymptomatic patients shows that approximately 19% of patients per year end-up with AVR due to onset of symptoms, which is quite similar to the annual AVR rate in the conservative arms of EARLY TAVR and EVOLVED (22.9% and 22.0%, respectively). The overall percentage of patients in RECOVERY who underwent AVR during follow-up was similar to that occurring in the other trials, but lower that might be anticipated considering that this is the trial with the longest follow-up and with the most severe degree of AS. One can speculate that the higher observed mortality in the control arm of AVATAR, and in particular the inflection point showing a steep increase in mortality after 5 years of follow-up in RECOVERY (Figure 1) was related to a more lenient clinical surveillance compared with the other trials. According to the study protocol, patients in the conservative arm of RECOVERY were evaluated clinically every 6 months, and an echocardiogram was performed every 12 months until 4 years after randomization. The increase in the death rate after 5 years coincides with the end of the required clinical and echocardiographic follow-up, suggesting less intense clinical surveillance occurring at that time point. Similarly, the death rate in the conservative arm of AVATAR challenges the statement that a significant proportion of patients in the conservative group of AVATAR during follow-up remained truly asymptomatic. At 5 years, mortality in the AVATAR conservative arm was almost 3 times higher than in the other trials. In the absence of signal of a higher incidence of SCD, it is possible that many of the deaths in AVATAR were related to unreported symptoms in patients erroneously classified as asymptomatic. Therefore, the survival benefit of AVR in truly asymptomatic patients might have been overestimated in both the surgical trials (RECOVERY and AVATAR). All evaluated trials have shown that SCD is quite uncommon in the control group, though this was previously a feared complication and justification to intervene earlier. Nonetheless, we need to highlight that these findings occur in a closely controlled cohort of patients typical of a control trial.

Studies have shown that almost 50% of patients with AS who undergo AVR have myocardial fibrosis demonstrable on cardiac magnetic resonance imaging and that the presence of fibrosis is associated with higher overall mortality after AVR (OR: 2.56 [95% CI: 1.83-3.57]).^10^ Data from the EVOLVED trial failed to show any benefit of early intervention (ie, before the onset of symptoms) in patients with myocardial fibrosis, but this occurred in the context of an admittedly underpowered trial. Mortality at 5 years in the conservative arm of EVOLVED was much higher than in EARLY-TAVR (approximately 20% vs 13.5%, respectively), suggesting that myocardial fibrosis is associated with worse prognosis in asymptomatic patients with AS.

The conservative arm of EARLY-TAVR featured the lowest annual death rate among all 4 trials (2.4%) and may explain the lack of statistical differences with the intervention group. Such a low mortality in the conservative arm may be due to the higher rate of AVR during the first year related to very close clinical surveillance.

Differences in baseline age across trials likely contributed to variations in overall mortality rates. These age-related differences may partly explain the higher early mortality observed in EVOLVED’s intervention arm and the lower absolute mortality in RECOVERY. However, the unexpectedly high mortality in AVATAR despite a younger population suggests that factors beyond age—such as intensity of surveillance and delayed AVR referral—may have also contributed.

Intervention arms: The overall death rate in the early intervention groups is generally quite similar among trials, but with exceptions. AVATAR reported a high short-term death rate, which plateaued after 1 year. Conversely, in EARLY TAVR, mortality was low during the first 2 years (1.8%/year), followed by a sharp increase after 4 years (approximately 5% to 6%/year) (Figure 1). There is a need for a longer-term follow-up to further define this trend. Although information from a landmark analysis in RECOVERY (looking at early and late follow-up benefits) would have been beneficial, early SAVR was associated with a benefit in overall mortality during the early stages of follow-up. This early benefit was mainly due to the 0% operative mortality in the surgical arm.

In the intervention arms of EVOLVED and EARLY-TAVR, overall mortality is very similar at 5 years, which highlights the benefit of early intervention even when there is evidence of myocardial fibrosis. An important difference between these 2 trials is that 75% of patients in the AVR arm of the EVOLVED trial underwent SAVR, while all patients underwent TAVR in EARLY TAVR. The PARTNER (Placement of Aortic Transcatheter Valves) 3 trial^11^ has shown that early mortality is higher after SAVR compared to TAVR in low-risk patients, but then mortality curves cross over after 4 years. Hence, the higher proportion of patients who underwent SAVR in the EVOLVED trial may explain the initial higher overall mortality in the early intervention group of this trial compared to EARLY TAVR.

### Major adverse cardiovascular events

#### Stroke

Most trials show a nonsignificantly lower risk of stroke with early AVR. In EARLY TAVR, the higher risk in the conservative group became more evident after 2 years of follow-up—a point of time when 70% of the conservative group had already undergone TAVR. Although there were no significant differences in the baseline demographic variables between the 2 arms of the trial, the most important numerical differences were related to the TAVR prosthesis used. In the early intervention arm, 81.3% received the SAPIEN 3 valve and 18.7% the SAPIEN ULTRA valve, while in the conservative arm, only 55.4% received SAPIEN 3, 40.2% received SAPIEN ULTRA, 1.8% a surgical valve, and 2.6% a nonstudy transcatheter heart valve. Unfortunately, there is no current information on the stroke risk according to each of these prostheses in the trial, nor on the antithrombotic treatment in these subgroups. Previous retrospective data had suggested that patients with severe AS may have a heightened risk of stroke and that AVR may decrease this risk.^12^ A trial-level meta-analysis of the current data from the 4 RCTs of asymptomatic AS supports this observation.^13^ Longer follow-up and subgroup analyses, further stratified by age and atrial fibrillation, are needed to further investigate this provocative finding.

#### Rehospitalization due to HF or CV causes

This outcome has been defined differently in each of the asymptomatic AS trials. Such heterogeneity in the definition of this outcome among trials makes it difficult to compare the benefit of early intervention across trials.

RECOVERY and AVATAR, however, used similar criteria (unplanned urgent admission due to HF) and reported a lower incidence of this outcome in the early intervention group (0% and 4%, respectively) compared to the conservative treatment group (11% and 17%, respectively). In EVOLVED, the incidence of AS-related hospitalization (a composite of unplanned admission due to HF, syncope, chest pain, heart block or ventricular arrhythmia) was also lower with early intervention (6% vs17%). In contrast, the incidence of unplanned hospitalization in EARLY TAVR was markedly divergent from the other 3 trials due to the different outcome definitions (AVR during the first 6 months was included as part of the definition). The incidence of unplanned hospitalization was 20.9% in the early intervention arm vs 41.7% in the conservative arm at a median follow-up of 46 months. At 6 months from randomization, 26.2% of the patients in the conservative group underwent aortic valve intervention, and these were therefore considered as components of the outcome.

The clinical need for AVR of nearly 1 in every 4 patients in the EARLY TAVR conservative group within 6 months of randomization in previously asymptomatic patients with a negative treadmill test seems higher than what is found in the literature regarding the natural history of AS. Additionally, the unplanned hospitalization rate in the early intervention arm of EARLY TAVR was higher than that reported in the TAVR arm of the PARTNER 3 trial (13.7% at 5 years), even though patients enrolled in PARTNER 3 had symptomatic AS.^11^ Considering the objective parameters used to recommend surgery in asymptomatic patients, out of the 116 patients from the conservative group who underwent AVR within the first 6 months only 2 had ≥3-fold increase in N-terminal prohormone of brain natriuretic peptide (NT-proBNP) and 5 had a LVEF decrease to <50% at the time of TAVR. Considering all patients of the conservative group who underwent TAVR during the follow-up (388 patients), 6% had ≥3-fold increase in NT-proBNP and 5% had LVEF <50% at the time of intervention.

It is known that patients and physicians in unblinded trials are subject to what has been labeled “subtraction anxiety”.^14^ Knowing that treatment was withheld in the control group, despite the computed tomography evaluation and heart team discussions indicating that all were candidates for TAVR, may cause anxiety in patients and/or in the referring physician, resulting in increased symptom awareness and the triggering of active treatment. Although this behavior is surely present in all the asymptomatic AS trials, its impact on the primary outcome is a major point of differentiation between EARLY TAVR and the other 3 RCTs.

Symptoms and quality of life: Evaluation of symptoms and quality of life (QOL) in open label trials is challenging due to the placebo effect and the aforementioned subtraction anxiety effect. EARLY TAVR evaluated symptoms and QOL using the Kansas City Cardiomyopathy Questionnaire (KCCQ). KCCQ scores were better in the early TAVR group during the course of the trial, as patients crossing over to TAVR in the conservative group had a transient decrease in QOL score at the time of crossover, but KCCQ scores were similar between the 2 groups at 2 years. Among patients in the conservative arm who crossed over to TAVR, the preprocedure QOL score was worse in patients who required AVR within 3 or 6 months than in patients who required intervention after 2 years of randomization. The decrease in the KCCQ score of almost 25 points in only 3 months after initial screening in asymptomatic patients suggests a strong effect of subtraction anxiety. Although KCCQ scores were similar in the 2 groups at 2 years, early intervention may avoid the transient worsening in QOL prior to the indication for TAVR.

With regard to symptoms, patients in the early AVR group had better NYHA functional class at follow-up in the EVOLVED trial, but no difference in objective parameters, such as development of LV systolic dysfunction, was found. LVEF was similar at follow-up between treatment arms in both EARLY TAVR and EVOLVED. Therefore, additional variables are required to assess symptom progression, especially in such open-label trials. Plasma levels of NT-proBNP and the 6-minute walk test distance have been previously used in intervention trials.^15^

In EARLY TAVR, “LV health” was evaluated through a composite outcome of LV global longitudinal strain ≥15%, a LV mass index <115 g/m^2^ for men or <95 g/m^2^ for women, and an left atrial volume index ≤34 mL/m^2^. LV health was present in 48.1% in the early intervention and 35.9% in the conservative group (*P* = 0.001) at the completion of follow-up. However, in both groups, all 3 parameters improved from baseline. Thus, early intervention is associated with better improvement in LV health, but a strategy of conservative treatment and intervention when symptoms appear is also associated with improvement. The precise meaning of improved LV health remains unclear, especially in the context of subtraction anxiety, and more information on its correlation with survival during longer-term follow-up is required.

## Interpretation of pooled data

Pooled study-level data from all 4 trials analyzed using a random effects model show that although early AVR is associated with lower stroke and unplanned hospitalization, overall and CV mortality are similar. The overall mortality forest plot of the recently published meta-analysis^13^ indicates that AVATAR was driving the nonsignificant trend to lower death rate with AVR. Considering the high mortality and low rate of SAVR in the conservative group, as previously noted, the mortality benefit of early SAVR reported in AVATAR should be interpreted with caution. Considering patient and treatment heterogeneity in these trials, as well as institutional-level outcomes (operative mortality), the optimal intervention strategy for patients with asymptomatic AS would require a patient-level meta-analysis separately for SAVR and TAVR. Until this information is available, close surveillance and frequent noninvasive evaluation of asymptomatic patients with severe AS appears to be associated with survival rates similar to those of early AVR (Central Illustration). Early TAVR is not associated with short- or mid-term adverse effects. However, data on long-term effects of early intervention with regard to long-term complications such as endocarditis and need for reintervention are necessary, which is especially important for younger patients with a longer life expectancy.

**Central Illustration:**
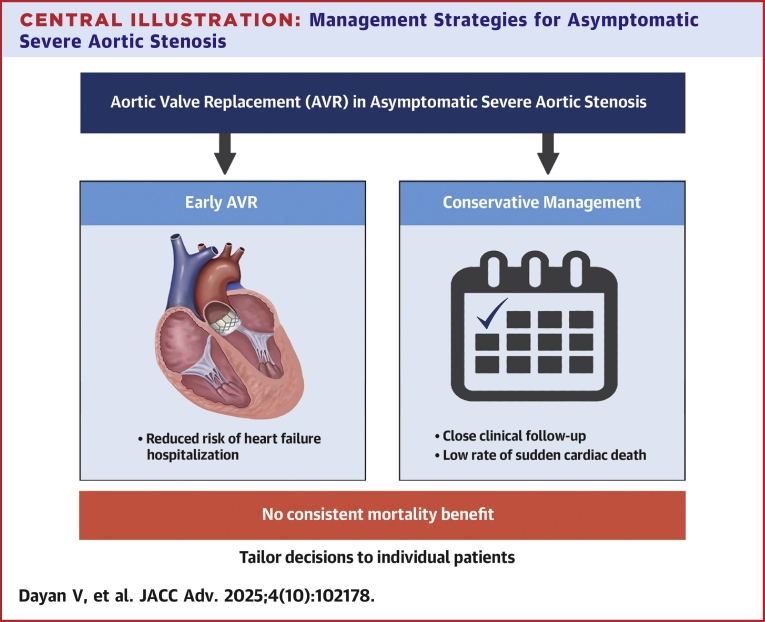
**Management Strategies for Asymptomatic Severe Aortic Stenosis** This schematic summarizes key findings from randomized trials evaluating early AVR vs conservative management in asymptomatic severe AS. Early AVR reduces heart failure hospitalizations but has not consistently shown a mortality benefit. Conservative management appears safe when combined with close clinical follow-up, as sudden cardiac death is rare in well-monitored patients. Clinical decision-making should be tailored to individual patient characteristics and preferences, balancing procedural risks, surveillance feasibility, and life expectancy. Abbreviations as in Figure 2.

## Future directions

The EASY-AS (Early valve replacement in severe ASYmptomatic Aortic Stenosis) trial (NCT04204915) is investigating early TAVR or SAVR vs surveillance in 2,844 asymptomatic patients with severe AS.^16^ The primary outcome of EASY-AS is CV mortality or HF hospitalization with results expected in 2031. PROGRESS (Management of Moderate Aortic Stenosis by Clinical Surveillance or TAVR) (NCT04889872) is randomizing 2,250 patients with moderate AS who have symptoms or evidence of cardiac damage or dysfunction to TAVR or surveillance with initial results expected in 2029. If PROGRESS shows benefit in moderate AS, it would provide supportive evidence that severe AS should be treated, even if asymptomatic. Individual patient-level meta-analyses of completed trials may also provide further insights.

## Conclusions

**Figure 2:**
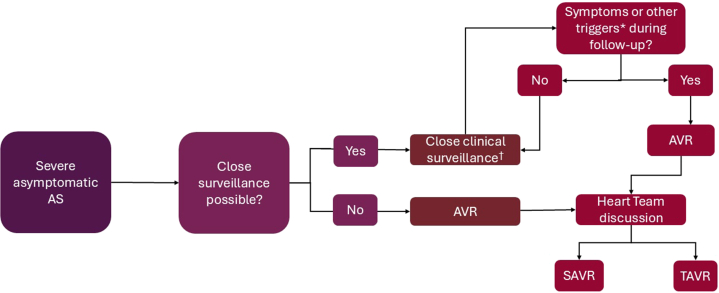
**Algorithm for Managing Patients With Asymptomatic Severe Aortic Stenosis** †Every 6 months. ∗Triggers: left ventricular ejection fraction ≤50%, positive exercise test, increased B-type natriuretic peptide (>3 times age- and sex-corrected normal range), very severe aortic stenosis (mean gradient >60 mm Hg or Vmax >5 m/s), severe valve calcification (assessed by cardiac computed tomography), Vmax progression 0.3 m/s/year. AS = aortic stenosis; AVR = aortic valve replacement; SAVR = surgical aortic valve replacement; TAVR = transcatheter aortic valve replacement.

Patients with asymptomatic severe AS require close clinical surveillance and periodic imaging and exercise stress testing. Current guidelines recommend patient education and clinical evaluations every 6 months in patients with severe AS, including serial echocardiograms to assess disease progression.^1^^,^^17^ In addition to echocardiographic assessment of valve hemodynamics and LVEF, it is reasonable to also measure global longitudinal strain. Longitudinal evaluation of exercise tolerance objectively with treadmill stress testing and serial measurement of circulating levels of natriuretic peptides should also be done at 6-month intervals. Early AVR may not affect mid-term survival but may be associated with fewer neurological events and more functional improvement. Conservative management is still reasonable, but only if there is patient awareness, frequent follow-up, and close clinical surveillance. AVR should be the treatment of choice if close clinical surveillance is difficult or not possible (Figure 2). Considering the low mortality in the conservative arms of the relevant trials, procedural risk of individual patients should be evaluated before considering either TAVR or SAVR. Shared decision-making is key in the decision to proceed with either TAVR or SAVR vs active surveillance.

## Funding support and author disclosures

Dr De Caterina has received fees, honoraria, and research funding from 10.13039/100004339Sanofi-Aventis, 10.13039/100001003Boehringer Ingelheim, 10.13039/100004326Bayer, BMS/10.13039/100004319Pfizer, 10.13039/501100022274Daiichi Sankyo, 10.13039/100004336Novartis, 10.13039/100004334Merck, Portola, 10.13039/100004337Roche, 10.13039/100004325AstraZeneca, Menarini, Guidotti, Milestone, Amarin, Noventure, and Amgen, all unrelated to this topic. Dr Van Mieghem has received institutional research grants from Abbott, Boston Scientific, Edwards Lifesciences, Medtronic, Meril, Pie Medical, PulseCath BV, and Teleflex; and has received consultancy fees from Abbott, Abiomed, Alleviant Medical Inc, AncorValve, Anteris, Approxima Srl, Bolt Medical, Boston Scientific, Daiichi Sankyo, LUMA Vision, Materialise, Medtronic, Pie Medical, Polares, PulseCath BV, and Siemens. Dr Borger declares that his hospital received speakers’ honoraria and/or consulting fees on his behalf from Edwards Lifesciences, Medtronic, Abbott, and Artivion. Dr Bhatt has served on advisory boards for Angiowave, Bayer, Boehringer Ingelheim, CellProthera, Cereno Scientific, E-Star Biotech, High Enroll, Janssen, Level Ex, McKinsey, Medscape Cardiology, Merck, NirvaMed, Novo Nordisk, Stasys; Tourmaline Bio; on the board of directors for American Heart Association New York City, Angiowave (stock options), Bristol Myers Squibb (stock), DRS.LINQ (stock options), and High Enroll (stock); has served as a consultant for Broadview Ventures, Corcept Therapeutics, GlaxoSmithKline, Hims, SFJ, Summa Therapeutics, and Youngene; has served on data monitoring committees for Acesion Pharma, Assistance Publique-Hôpitaux de Paris, Baim Institute for Clinical Research (formerly Harvard Clinical Research Institute, for the PORTICO trial, funded by 10.13039/100006279St. Jude Medical, now Abbott), 10.13039/100008497Boston Scientific (Chair, PEITHO trial), Cleveland Clinic, Contego Medical (Chair, PERFORMANCE 2), Duke Clinical Research Institute, Mayo Clinic, Mount Sinai School of Medicine (for the ENVISAGE trial, funded by Daiichi Sankyo; for the ABILITY-DM trial, funded by Concept Medical; for ALLAY-HF, funded by Alleviant Medical), Novartis, Population Health Research Institute; Rutgers University (for the NIH-funded MINT Trial); has received honoraria from American College of Cardiology (Senior Associate Editor, Clinical Trials and News, ACC.org; Chair, ACC Accreditation Oversight Committee), Arnold and Porter law firm (work related to Sanofi/Bristol Myers Squibb clopidogrel litigation), Baim Institute for Clinical Research (formerly Harvard Clinical Research Institute; AEGIS-II executive committee funded by 10.13039/100008322CSL Behring), Belvoir Publications (Editor in Chief, Harvard Heart Letter), Canadian Medical and Surgical Knowledge Translation Research Group (clinical trial steering committees), CSL Behring (AHA lecture), Cowen and Company, Duke Clinical Research Institute (clinical trial steering committees, including for the PRONOUNCE trial, funded by 10.13039/501100004914Ferring Pharmaceuticals), HMP Global (Editor in Chief, *Journal of Invasive Cardiology*), *Journal of the American College of Cardiology* (Guest Editor; Associate Editor), Level Ex, Medtelligence/ReachMD (CME steering committees), MJH Life Sciences, Oakstone CME (Course Director, Comprehensive Review of Interventional Cardiology), Piper Sandler, Population Health Research Institute (for the COMPASS operations committee, publications committee, steering committee, and USA national co-leader, funded by Bayer), WebMD (CME steering committees), Wiley (steering committee); other: Clinical Cardiology (Deputy Editor); patent: Sotagliflozin (named on a patent for sotagliflozin assigned to Brigham and Women's Hospital who assigned to Lexicon; neither I nor Brigham and Women's Hospital receive any income from this patent); has received research funding from Abbott, Acesion Pharma, Afimmune, Aker Biomarine, Alnylam, Amarin, Amgen, AstraZeneca, Bayer, Beren, Boehringer Ingelheim, Boston Scientific, Bristol Myers Squibb, Cardax, CellProthera, Cereno Scientific, Chiesi, CinCor, Cleerly, CSL Behring, Faraday Pharmaceuticals, Ferring Pharmaceuticals, Fractyl, Garmin, HLS Therapeutics, Idorsia, Ironwood, Ischemix, Janssen, Javelin, Lexicon, Lilly, Medtronic, Merck, Moderna, MyoKardia, NirvaMed, Novartis, Novo Nordisk, Otsuka, Owkin, Pfizer, PhaseBio, PLx Pharma, Recardio, Regeneron, Reid Hoffman Foundation, Roche, Sanofi, Stasys, Synaptic, The Medicines Company, Youngene, and 89Bio; has received royalties from Elsevier (Editor, Braunwald’s Heart Disease); and has served as site co-investigator for Cleerly. All other authors have reported that they have no relationships relevant to the contents of this paper to disclose.
